# Mechanism and Antibacterial Activity of Gold Nanoparticles (AuNPs) Functionalized with Natural Compounds from Plants

**DOI:** 10.3390/pharmaceutics14122599

**Published:** 2022-11-25

**Authors:** Anna Timoszyk, Renata Grochowalska

**Affiliations:** 1 Laboratory of Biophysics, Department of Biotechnology, Faculty of Biological Sciences, University of Zielona Góra, Szafrana 1, 65-516 Zielona Góra, Poland; 2 Laboratory of Biochemistry and Cell Biology, Department of Biotechnology, Faculty of Biological Sciences, University of Zielona Góra, Szafrana 1, 65-516 Zielona Góra, Poland

**Keywords:** biosynthesis, gold nanoparticles, plant extract, antibacterial activity, antibacterial mechanism, reduction potential

## Abstract

Recently, the biosynthesis of gold nanoparticles (AuNPs) has been widely studied and described. In the age of bacterial drug resistance, an intensive search for new agents with antibacterial properties or a new form of antibiotics with effective action is necessary. As a result, the antibacterial activity of AuNPs functionalized with natural compounds is being investigated more frequently. AuNPs biosynthesized with plant extract or functionalized with bioactive compounds isolated from plants could be particularly useful for pharmaceutical applications. The biosynthesized AuNPs are stabilized by an envelope, which may consist of flavonoids, phenolic acids, lipids and proteins as well as carbohydrates and vitamins. The composition of the natural coating affects the size, shape and stability of the AuNPs and is also responsible for interactions with the bacterial cell wall. Recently, several mechanisms of AuNP interactions with bacterial cells have been identified. Nevertheless, they are not yet well understood, due to the large diversity of plants and biosynthesized AuNPs. Understanding the antibacterial mechanisms allows for the creation of pharmaceutical formulations in the most useful form. Utilizing AuNPs functionalized with plant compounds as antibacterial agents is still a new concept. However, the unique physicochemical and biological properties of AuNPs emphasises their potential for a broad range of applications in the future.

## 1. Introduction

Nanoparticles (NPs) can be obtained by various methods, and the increased interest in metallic NPs (MNPs) has forced the development of new synthesis strategies that are inexpensive, easy to carry out, and most importantly, environmentally friendly [1]. Recently, there has been increased interest in biological methods of obtaining MNPs. Unlike chemical syntheses, biological methods do not use toxic reducing, blocking and stabilizing compounds, which makes biosynthesis eco-friendly [2]. Moreover, the obtained NPs are biocompatible. On the other hand, physical methods require specialized equipment and large amounts of energy to carry out the synthesis process (e.g., generation of high pressure and temperature, ultrasound waves, UV radiation, etc.), which makes the synthesis process time-consuming and expensive [3].

MNPs, including gold NPs (AuNPs), are of great interest due to their intrinsic surface plasmon resonance (SPR) property. The SPR of AuNPs promotes their use in imaging diagnostics, anti-cancer therapy to induce local hypothermia, or as biosensors and biomarkers [4,5]. Additionally, AuNPs are highly valued for their unique biological properties such as biocompatibility, facile surface functionalization, catalytic activity and the ability to reveal cytotoxic and/or antimicrobial activity [5]. AuNPs having various types of envelopes, i.e., surfaces functionalized with active biomolecules which give them unique properties, are especially useful for antimicrobial applications [6,7,8]. Currently, the most widespread searches regarding AuNPs are those for specific biological properties. Biosynthesized AuNPs, which have specific biological properties due to the natural envelope formed during the biosynthesis process, are included in these searches [9]. Products of natural origin are used for the biosynthesis of AuNPs; most often, microorganisms and plants are used [3]. Biological methods for the synthesis of AuNPs are more attractive than conventional methods due to the greater availability and variety of the material used. In addition, the waste generated during ingredient preparation and post-reaction does not have a negative impact on the environment, and it is easier and cheaper to dispose of biosynthesis waste compared to the waste generated using conventional methods [3,10,11]. Recently, great progress has been made regarding the biological activity and the possible applications of AuNPs synthesized using plant extracts or substances isolated from them [10,12,13]. Due to this trend, the number of publications related to the synthesis of AuNPs from plant material has increased. The biological activity of the plant extracts themselves and the compounds they contain, as well as the vast variety of research material, have led to the increased acceptance of biosynthesis as a promising method to obtain AuNPs [4,7,13].

Plants have the ability to synthesize NPs both intracellularly and extracellularly. Intracellular methods of synthesizing NPs include culturing plants in metal-rich organic environments, e.g., metal-rich soil or hydroponic solutions. This method of obtaining NPs is usually aimed at applications outside of the biomedical space. In contrast, extracellular methods include the NPs synthesized using an extract obtained from the leaves, flowers, fruits or other parts of the selected plant [14]. Interestingly, extracts from different parts of the same plant can significantly differ in their composition and biological properties. Bioactive compounds present in plant extracts are primarily flavonoids, phenols, citric acid, ascorbic acid, polyphenols, terpenoids and alkaloids [9,12,14]. Many substances belonging to these groups of compounds have antioxidant properties and the ability to reduce gold ions to metallic gold.

**Figure 1:**
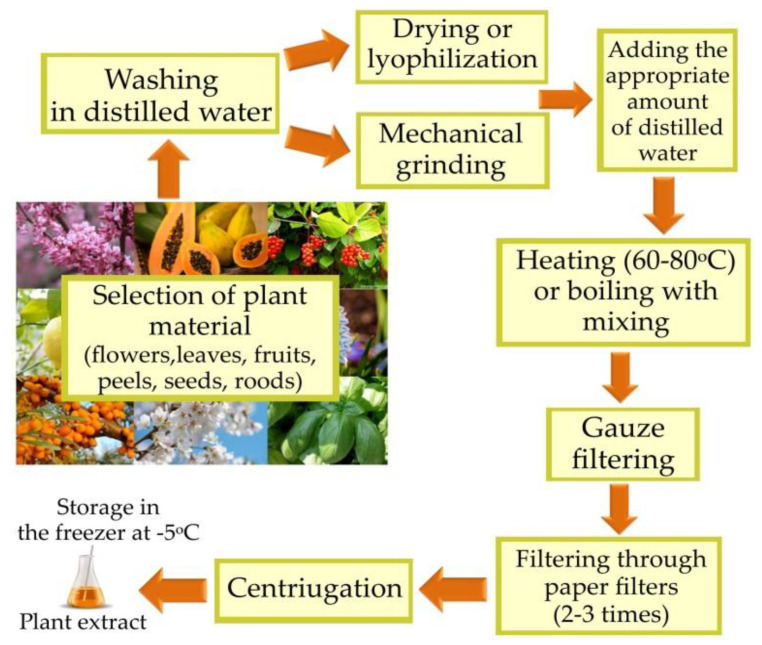
Preparation of plant extract.

The biosynthesis of AuNPs from plant extracts is an easy process. The selection of the plant from which the extract will be prepared is important because the plant species and the part from which the extract will be obtained affect the amount of reducing compounds and the formation of the envelope coating around the AuNPs (Figure 1) [1,15,16].

First, the harvested plant material is thoroughly rinsed in distilled water. The next step is mechanically grinding the material with the addition of distilled water. Then, the mixture is heated to 60–80 °C or, in some cases, it is brought to the boil. Afterward, the obtained mixture is filtered several times. Finally, the filtrate is centrifuged [9]. The aqueous plant extract prepared in this way can be used for the biosynthesis of AuNPs or stored in a refrigerator (at −5 °C) until needed. The second reactant of the reaction is an aqueous solution of chloroauric acid (HAuCl_4_), which should be added in an appropriate amount to the prepared plant extract. The most commonly used concentrations of HAuCl_4_ solution range from 0.5–30 mM. The biosynthesis process is carried out in the dark at an appropriate temperature ranging from 25–90 °C [7,11]. The successful synthesis of AuNPs is evidenced by the color change of the mixture from yellow to pink, dark red or purple [17]. Particular attention is paid to the influence of physicochemical factors, i.e., concentration of reaction substrates, pH, temperature and duration of reaction, on the resultant NPs of the biosynthesis process. By selecting the appropriate synthesis conditions and the appropriate plant extract, the size and shape as well as the rate of formation and stability of AuNPs can be controlled [18]. Moreover, biosynthesized AuNPs can be complexed with other nanostructures or functionalized in a way to enhance their properties and increase their scope of application [19,20].

AuNPs are biocompatible, so they can easily bind to proteins or nucleic acids [21]. Biofunctionalized AuNPs with biologically active molecules incorporated within the NP envelope have recently become a popular subject of research due to their specific properties and their potential application in many areas, including as antibacterial agents [22]. Developing the most effective methods of fighting pathogens and preventing and treating bacterial diseases is a primary goal of current research [23]. The need for new antibacterial agents is due in part to the increasing resistance of bacteria to known antibiotics, but also due to the desire to increase the drug potency while reducing side effects [24]. Therefore, scientists aim to develop a broad acting antimicrobial agent which will reduce side effects to a minimum, and at the same time will not be toxic to bacteria that do not cause diseases but form bacterial flora of humans, such as *Lactobacillus* [23]. Notably, biosynthesized AuNPs would be advantageous as a new antibacterial agent because synthesized bacteria are unable to acquire resistance to them [25].

Studying the antibacterial activity of biosynthesized AuNPs is relatively complicated due to the multitude of parameters on which it depends, including the physicochemical conditions of the synthesis reaction (on which the shape and size of the AuNPs depend), the composition of the AuNPs’ envelope (on which the surface charge and stability of the AuNPs depend), the specificity of the interaction with bacterial cells at the molecular level, and parameters common to all pharmaceuticals (i.e., the type of bacteria and the concentration of AuNPs) [26,27]. Two terms are used to define the antimicrobial properties of tested substances: bacteriostatic and bactericidal. Bacteriostatic agents delay the growth of bacteria and stop their initial growth phase for a long time. Bactericidal agents completely inhibit bacterial growth [28,29]. The methods used to determine the antibacterial activity of other agents are also used to evaluate the antimicrobial activity of biosynthesized AuNPs. The most common method is the disk-diffusion method (zone of inhibition) and determining of the minimum inhibitory concentration (MIC_50%_) value, which is the minimum amount of a substance that inhibits the growth of 50% of bacteria, using the dilution method. The antibacterial activity of biosynthesized AuNPs differs depending on the type of bacteria, which is mainly related to the difference in the structure of the cell walls of Gram-positive and Gram-negative bacteria. Gram-negative bacteria have a more complex cell wall in terms of structure, but it is much thinner (from 2 to 10 nm) and much more susceptible to damage compared to the cell walls of Gram-positive bacteria [30]. The cell walls of Gram-negative bacteria contain one layer of murein (peptidoglycan) between two lipid membranes. The outer membrane consists of phospholipids, proteins and lipopolysaccharides [31]. The cell wall thickness of Gram-positive bacteria varies from 15 to 80 nm. The cell walls of Gram-positive bacteria are devoid of the outer lipid membrane, and the densely cross-linked murein contains teichoic acids, proteins and lipids [30,31].

Bacteria have developed mechanisms for protection and acquiring resistance. Therefore, it is important to learn about the specificity of the interaction of biosynthesized AuNPs with bacterial cells, determine the parameters on which the antibacterial activity of AuNPs depends, and identify what the mechanism of action of AuNPs on bacteria is and the path of cell death. The presented review aims to discuss the current knowledge of AuNPs synthesized from plant extracts for use as antibacterial agents while focusing on the suitability of plant products for the biosynthesis of AuNPs, the antibacterial properties of the biosynthesized AuNPs and plant extracts, and the parameters on which the antibacterial activity of biosynthesized AuNPs depends. Analyzing these research results will lead to conclusions regarding how biosynthesized AuNPs interact with bacterial cells and the potential mechanisms of their antibacterial action.

## 2. Biosynthesis of AuNPs Using Plants

### 2.1. Reduction Potential of Plant Extracts

Plants are a source of biologically active compounds that not only possess antioxidant properties, but also act as reducing agents in the biosynthesis reaction [8,11,12,32]. The total reduction capacity of plant extracts can be determined by studying electron transfer with antioxidants by reaction with the Folin–Ciocalteu reagent, the DPPH radical, or using one of the electrochemical methods [33,34]. The reduction potential of plant extracts may vary significantly depending on the composition of bioactive compounds [1,3,35]. Well-known reducing substances include secondary metabolites of plants such as sugars, terpenoids, polyphenols, alkaloids and proteins, most of which possess antioxidant properties [1,15,36] (Table 1). The in vitro antioxidant activity and reduction potential of *Crassocephalum rubens* leaf extract were investigated, and the obtained AuNPs synthesized using this extract were deemed suitable for future applications. The antioxidant potential of the post-reaction mixture was lower than that of the extract itself. Thus, the obtained results indicate that substances with antioxidant properties are associated with the reduction of gold ions to metallic gold [37]. The most common antioxidants in plant extracts are terpenoids and polyphenols. Terpenoids are a group of organic polymers that consist of five-carbon isoprene units. In contrast, flavonoids are a large group of polyphenolic compounds that includes anthocyanins, isoflavonoids, flavonols, chalcones, flavones, and flavanones [38,39]. Terpenoids and flavonoids can actively reduce metal ions to NPs because they contain various functional groups [40,41]. Additionally, monosaccharides can play a reducing role. Monosaccharides that contain a ketone group can act as an antioxidant only when the ketone is converted tautomerically into an aldehyde [40,41,42]. Moreover, the reducing capacity of disaccharides and polysaccharides depends on the composition of the monosaccharides and their ability to share an aldehyde group with the metal ion. Amino acids such as lysine, cysteine, tryptophan, tyrosine, arginine and methionine possess a high ability to bind various metal ions and reduce them [40,43].

Thus, the process of AuNP synthesis from plant extracts is closely related to the reduction potential and the presence of appropriate functional groups [44]. Three factors have a clear impact on biosynthesis efficiency. These include: the degree of reduction by metal ions (reduced by individual substances contained in the extract), the concentration of the reducing compounds and the composition of bioactive compounds forming the envelope stabilizing the AuNP [18]. Greater total content of reducing substances in the plant extract accelerates AuNP formation, increases the fraction of small NPs and increases the stability of the AuNPs [12,18,45]. The efficiency of the MNP biosynthesis process also depends on the electrochemical potential of a given metal ion [40]. The reduction potential of all noble metal salts ranges from 0.35 to 1.0 V. Any type of metal ions can be reduced to MNPs, provided that the reduction potential of the extract is greater than +0.16 V [44]. Hossanisaadi et al. (2021) screened and compared studies of plant extracts in terms of their ability to reduce gold ions. The review presents the results regarding the ability of the extracts from 27 plants, including *Rosa damascena*, *Juglans regia*, *Caccinia macranthera*, *Urtica dioica*, *Areca catechu* and *Anethum graveolens*, which are used in traditional medicine in the Middle East, to reduce gold ions. Extracts were prepared from various parts of the plants. Additionally, 28 new plants with suitable extracts were also identified. The derived extracts were able to successfully reduce gold ions during biosynthesis [46].

Plant extracts, especially fruit extracts, contain high concentrations of reducing compounds. For example, blackberries, blueberries, grapes, *Citrullus lanatus*, *Cornus mas*, *Punica granatum*, and *Terminalia arjuna* extracts contain large amounts of flavonoids, phenolic compounds, anthocyanins, saccharides, ascorbic acid and other vitamins [47,48,49,50,51]. Biosynthesis carried out using the extracts from these plants is more effective and less expensive compared to traditional chemical synthesis due to the abundance and presence of naturally occurring reducing agents [9]. In order to investigate the reducing properties of the *Papaver somniferum* extract, the synthesis of AuNPs was carried out. Moreover, they discovered that methanol extract has a high reducing potential with a high affinity for gold cations. The resulting spherical AuNPs were 77 nm in diameter and stabilized by phytochemicals present within the extract [52]. On the other hand, the synthesis of AuNPs carried out with the use of *Artemisia capillaris* extract showed that the composition of the plant extract had a significant impact on biosynthesis. The extract contained saponins, amino acids, phenolic compounds, flavonoids and diterpenes, but only flavonoids, phenolic compounds and amino acids were involved in the synthesis of AuNPs [53,54]. Examination of the composition of amino acids in the lyophilized extract of *Galaxaura elongata* revealed the presence of glutamic acid, asparagine, leucine, lysine, glycine and alanine. Amino acids were responsible for the reduction and stabilization of AuNPs, but sulphate polysaccharides and polypeptides also played a role [55]. Presumably, the reduction of gold ions by amino acids is due to the hydroxyl and carboxyl groups [56].

The influence of the extract composition, particularly the reducing compounds present in the extract, impact the size of AuNPs generated via biosynthesis. During the synthesis of AuNPs with ethanolic extract from black tea, the tannins acted as a reducing and stabilizing agent [57]. Spherical NPs were obtained with a bimodal size distribution of 10 nm and 3 nm for two fractions of AuNPs. Similarly, AuNPs synthesized using the *Plumeria alba* extract also resulted in a bimodal size distribution with spherical NPs either in the range of 20–30 nm or 80–150 nm [57]. Spherical AuNPs synthesized with fruit infusion from *Medinilla speciosa* were 200–450 nm in diameter and the phenolic compounds were responsible for the reduction of gold ions and stabilization of the AuNPs [36]. During the synthesis of AuNPs using the *Mimosa tenuiflora* extract, the *v*/*v* ratio of the reagents had a significant impact on the biological activity of the obtained AuNPs. However, neither the size of the AuNPs nor the composition of the plant extract were found to impact the biological activity of the resulting AuNPs [10].

Plants produce numerous secondary metabolites with antioxidant properties and enzymes that prevent oxidative damage to cell organelles and their contents. Flavonoids, flavonoid glycosides and vitamins, such as ascorbic acid isolated from plant extract, have also been shown to reduce gold ions [58]. Leaf extracts from medicinal plants are used for most of the AuNPs synthesis reactions. Active herbal compounds such as polyphenols are involved in the reduction of gold ions and the stabilization of AuNPs [54,59]. Active compounds isolated from the extract of *Ocimum sanctum*, such as apigenin, cirsimaritin, rosmarinic acid, estragole, linalool, carvacrol and urosolic acid, have numerous pharmaceutical applications, and the ligands of these compounds can reduce metal ions [60]. In the case of AuNPs synthesized using fruit extract from *Genipa americana*, substances such as genipin, genipaol, geniposide and ranolazine acted as reductants of gold ions. During AuNP synthesis using the extract of *Lycopersicon esculentum*, citric and ascorbic acids also had the ability to reduce gold ions [61,62].

AuNPs of various sizes were synthesized using pectins isolated from *Musa paradisiaca* fruit extracts and orange peels [63,64,65,66,67,68,69,70]. The resultant AuNPs were biocompatible with bacterial cells, cytotoxic against HeLa and HepG2 cell lines and zebrafish embryos, and showed anti-inflammatory activity [68,69,70]. Curcumin isolated from *Curcuma longa* was also investigated as a reducing and stabilizing agent for AuNPs. Curcumin is well known and primarily investigated due to its anti-cancer properties. Moreover, many research teams have successfully used curcumin to synthesize AuNPs under various pH and temperature conditions [71].

**Table 1:** Compounds responsible for creating the reducing potential of plant extracts.

Compounds	Plant	Kind of Extract	References
Phenolic compounds, flavonoids	*Crassocephalum rubens*	Leaf; water extract	[37]
Anthocyanins	*Cornus mas*	Fruit; water extract	[47]
Anthocyanins	*Punica granatum*	Fruit; water extract	[48]
Cholidonic, superbine, colchicine, gloriosol, phytosterils and stigmasterin	*Gloriosa superba*	Leaf; water extract	[49]
Pectins, ribose, phenolic compounds	*Papaver somniferum*	Leaf; methanol extract	[52]
Amino acids, phenolic compounds, flavonoids	*Artemisia capillaris*	Whole plant; water extract	[53,54]
Glutanic acid, asparagine, leucine, lysine, glycine, alanine	*Galaxaura elongata*	Whole plant; water extract	[55,56]
Tannins	Black tea	Leaf; ethanol extract	[57]
Phenolic compounds	*Medinilla speciosa*	Fruit; water extract	[36]
Catechins, ascorbic acid	*Mimosa tenuiflora*	Tree bark; water/ethanol extract	[10]
Estragole, linalool, carvacral, urosalic acid, cirsimarin, rosmarinic acid	*Ocinum sanctum*	Flower and leaf; water extract	[60]
Genipin, genipol, geniposide, ronolazine	*Genipa americana*	Fruit; water extract	[61]
Citric and ascorbic acid	*Lycopersicon esculentum*	Fruit; water extract	[62]
Pectins	*Musa paradisiaca*	Fruit; water extract	[63,64,65,66,67,68,69,70]
Pectins	*Citrus sinensis*	Peels; water extract	[63,64,65,66,67,68,69,70]
Curcumin	*Curcuma longa*	Water solution	[71]

### 2.2. Mechanism of AuNPs Biosynthesis Using Plants

**Figure 2:**
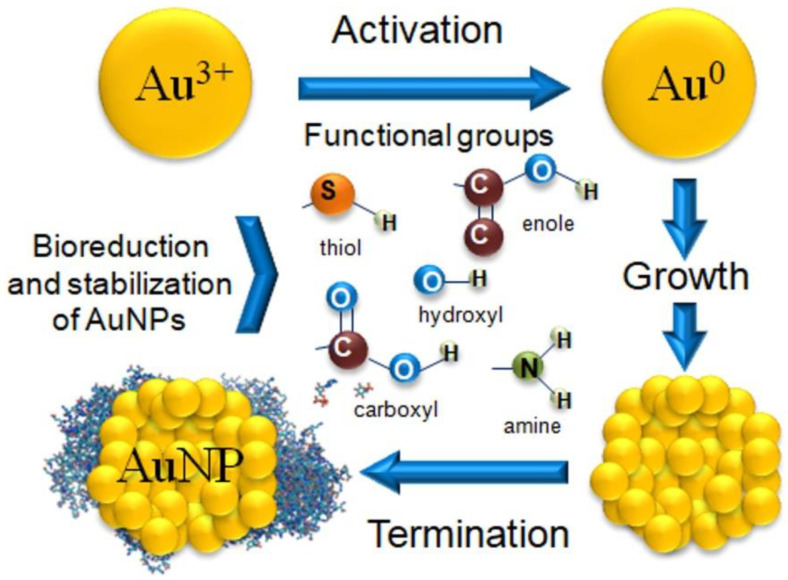
Mechanism of AuNPs biosynthesis and functional groups involved in the reduction of gold ions and stabilization of AuNPs.

The biosynthesis of MNPs can take place through biogenesis and bioreduction. Biogenesis utilizes microorganisms. In contrast, only bioreduction is possible when using plant extracts or substances isolated from plants [72]. The process of AuNPs biosynthesis begins with the reduction of gold ions, i.e., activation, which depends on the reducing potential of the extract. The next stage of biosynthesis is the growth of the NPs [63,73]. This process involves increasing the size of the NP nuclei (seeds) and the merging of the NP nuclei into clusters. The last stage of the process, i.e., termination, continues until thermodynamic equilibrium is achieved and results in the formation of the final shape and size of the AuNPs (Figure 2) [18,73].

The shape and size of AuNPs are influenced by electrostatic interactions between bioactive compounds derived from the plant extract and metallic gold [74]. The source of gold ions is the HAuCl_4_ solution in which three Cl atoms are covalently bonded and the fourth is coordinated. Many studies show that the mechanism of the reduction reaction depends on the number of Cl ligands in the metal complex, which is the source of the energy differences during the reaction [75]. For example, the synthesis of AuNPs was performed using 1,8-cineole obtained from the extract of *Eucalyptus*, an organic compound belonging to the terpenes. They discovered that the oxidation of 1,8-cineole initiated the entire biosynthesis process. Thus, the presence of a water molecule was necessary for energy reduction, and the bioreduction process itself took place in several stages [76].

The presence of hydroxyl or amino groups in the plant extract play an important role in the process of reducing gold ions to metallic gold. This process can take place during an oxidation reaction or due to the formation of specific quinine forms [43,60]. Gold reduction has also been demonstrated during the tautomeric conversion of flavonoids (from the enol form to the ketone form). In this reaction, a reactive hydrogen atom is released which can reduce gold ions to metallic gold [43]. The internal mechanism of the transformation of flavonoids from ketones to carboxylic acids may also be responsible for the reduction of gold ions [9]. In the case of AuNP synthesis using *Garcinia cambogia* and *Pyrus* fruit extracts, saccharides acted as a reducing agent. The reduction of gold ions most likely involves the oxidation of an aldehyde group to a carboxyl group by nucleophilic addition of a hydroxyl group [77,78]. Numerous plant extracts contain proteins with reducing potential. However, a protein’s ability to reduce gold ions varies based on its amino acid sequences [79].

### 2.3. Conditions of Biosynthesis Reaction

Physicochemical parameters have a significant impact on the course of each reaction, including biosynthesis, affecting the rate of the reaction and the size, shape and stability of the obtained AuNPs [18,35]. Moreover, the concentration of reactants, temperature, pH and the duration of the reaction have a decisive influence on the products of biosynthesis [80].

#### 2.3.1. Role of the Reactant Concentration

The concentration of reactants or the *v*/*v* ratio of reactants influences the size and shape of AuNPs, as well as the duration of the biosynthesis. AuNP biosynthesis from *Solanum indicum* fruit extract was more effective when the concentration of the fruit extract and/or chloroauric acid solution was increased [81]. In the AuNP biosynthesis process using *Phyllanthus amarus*, the concentration of the extract itself played a key role. When the extract concentration was too low, AuNPs of various shapes were formed. On the other hand, at higher concentrations of extract, spherical AuNPs were formed [82].

At the lowest investigated concentration of the *Artemisia capillaris* extract, the duration of AuNPs biosynthesis was 1 h. However, at the highest extract concentrations, the biosynthesis reaction decreased to 30 min. Additionally, the size of the AuNPs decreased with increasing concentration of extract [53]. The optimal reaction time for the biosynthesis of AuNPs carried out using the *Padina tetrastromatica* extract was also investigated. Moreover, this reaction lasted 24 h resulting in small, spherical AuNPs [27].

AuNPs synthesized using *Carallia brachiata* leaf extract and at HAuCl_4_ solution concentrations greater than 1 mM increased the reaction rate and decreased the stability of AuNPs. However, increasing the volume of extract decreased the size of the obtained AuNPs [83]. Decreased NP stability due to an increased concentration of HAuCl_4_ solution was also observed for AuNPs obtained using *Elaeis guineensis* leaf extract [84]. At a concentration of HAuCl_4_ solution greater than 0.5 mM, the size of AuNPs synthesized using *Solidago canadensis* leaf extract increased dynamically (up to 250 nm) and NPs formed various shapes. Interestingly, in this case, an inverse relationship was observed between the rate of biosynthesis and the concentration of reagents [85].

#### 2.3.2. Role of pH

The pH of the reaction medium is of particular importance because this parameter determines whether the reaction will take place at all and, ultimately, what shape and size the AuNPs will be. The reaction pH affects the reducing compounds in the extract, and thus changes their charge [40]. Depending on the pH of the reaction, differences in the size of the obtained AuNPs were also observed [9]. The influence of the reaction pH on the size of AuNPs was investigated during biosynthesis with *Mangifera* peel extract. The more alkaline the pH was, the smaller the AuNPs were. In an alkaline pH of 9, AuNPs were 6 nm; in an acidic pH of 2, AuNPs were three times larger [3]. A similar relationship was found in the case of AuNPs synthesized using *Carallia brachiata* leaf extract. Additionally, as the pH increased, the AuNPs became less polydisperse [83]. Larger AuNPs are usually formed in an acidic pH environment and smaller AuNPs form in an alkaline pH [86]. This relationship is most likely due to the electrostatic interaction between gold ions and the functional groups of reducing compounds. Under acidic conditions, biosorption is enhanced. On the other hand, an increase in pH (i.e., a decrease in the concentration of protons in the solution) drastically reduces the biosorption potential of the extract. This is likely because the lower pH neutralizes the negative charge of the functional groups of biomolecules, which increases the intermolecular attraction [87]. An inverse relationship was also observed between reaction pH and the size of the resultant AuNPs. At a pH of 3.2, the most spherical AuNPs were formed in the NP biosynthesis using *Padina tetrastromatica* extract. For reaction carried out in the pH range of 7–10, AuNPs were polydisperse and polymorphic [27]. A similar relationship was observed for AuNPs synthesized with the extract from the stem of *Periploca aphylla*. Additionally, at a pH of 4, the smallest AuNPs formed [88]. AuNPs obtained using *Pyrus* extract were also the most polydisperse at alkaline pH [78]. Therefore, the acidic pH was optimal for these biosynthesis reactions.

#### 2.3.3. Role of Temperature

Increasing reaction temperature above room temperature will increase the rate of synthesis and makes the reaction more effective. Temperature may also affect the shape and size of the formed AuNPs due to the shortened total reaction time. The biosynthesis of AuNPs from *Padina tetrastromatica* was carried out at three temperatures: 25 °C, 60 °C and 90 °C. An increase in AuNP size was observed at temperatures above room temperature, at which point monodisperse and spherical NPs were obtained [27]. Temperature was also found to influence the shape of AuNPs synthesized using *Cassia fistula* extract. Nanotubes were formed mainly at room temperature, whereas spherical AuNPs were formed at temperatures above 60 °C [40]. During the synthesis of AuNPs using *Magnolia kobus* leaf extract, an inverse relationship was observed between the size of the obtained NPs and the reaction temperature. As a result, syntheses carried out at 25 °C produced large AuNPs, whereas small AuNPs were obtained when the same experiment was run at 95 °C [60].

## 3. Antibacterial Activity of AuNPs Biosynthesized from Plants

The antibacterial activity of AuNPs biosynthesized from plant extracts has been an important research topic for a long time due to the unique physicochemical and biological properties that make them suitable for use as antibacterial agents [89]. Antibacterial activity is mainly attributed to a high surface-to-volume ratio, and the small size of AuNPs facilitates their penetration into cell walls and membranes [35,90]. The dependence of antibacterial activity on the size of AuNPs and their concentration was initially confirmed using bare, chemically synthesized AuNPs [91,92,93]. Many studies using biosynthesized AuNPs suggest that positive antibacterial tests are due to the composition of the AuNP envelope formed during the bioreduction process, the surface charge and the stability of AuNPs. The presence of non-reduced Au^1+^ and Au^3+^ ions is also important [36,89,94,95]. This effect was noticed, for example, in the case of AuNPs synthesized from *Ziziphus zizyphus* leaf extract, which at a concentration of 5 mg/mL had no effect on Gram-negative *E. coli*, while gold ions did [58]. This is because gold ions are toxic and cytotoxic [96]. Unreduced gold ions may also be present in the post-reaction mixture as a result of incorrect selection of the *v*/*v* ratio of the reagents, or the fact that the synthesis reaction is not yet complete [14]. Biosynthesized AuNPs may have antibacterial properties and may be active only against Gram-positive or Gram-negative bacteria, or show antibacterial properties simultaneously against both types of bacteria (Table 2) [97].

The antibacterial activity of biosynthesized AuNPs is usually concentration-dependent, and the lack of such a relationship is very rare. For example, AuNPs biosynthesized by Srinath (2017) showed activity against the Gram-positive bacteria *S. aureus*, regardless of the concentration. However, in the case of *E. coli* Gram-negative bacteria, the antibacterial activity was observed only at the concentration of AuNPs equal to 100 µL/mL [97].

The only difference was the reducing biomaterial used to carry out the reaction. In the first case, secondary metabolites of plants were used. In the second example, secondary metabolites of bacteria were utilized, which explains the influence of the type of bioreducer used on the antibacterial properties of AuNPs. The antibacterial activity of AuNPs synthesized with extracts from *Ocimum tenuiflorum* flowers and leaves, *Azadirachta indica* and *Mentha spicata* leaves and *Citrus sinensis* peels was also investigated [15]. At a concentration greater than 512 µg/mL, all biosynthesized AuNPs showed activity against Gram-positive *S. aureus* and Gram-negative *P. aeruginosa* and *K. pneumoniae*. The dependence of antibacterial activity of biosynthesized NPs on their concentration was also confirmed using AuNPs obtained using the extract from *Annona muricata* leaves which showed activity against Gram-positive and Gram-negative bacteria (*C. sporogenes*, *S. aureus*, *E. faecalis* and *K. pneumoniae*), AuNPs synthesized using *Azadirachta indica* leaf extract and *Zingiber officinale* root which were active against *E. faecalis*, *S. mutant* and *S. aureus*, and many other AuNPs biosynthesized using plants [114,119]. In addition, each of these plant extracts had a different composition, which influenced the reduction of Au^3+^ ions to Au^0^ and the size and stabilization process of AuNPs [15]. In a few cases, no dependence of the antibacterial activity on the concentration of AuNPs was observed; for example, in the case of AuNPs synthesized using *Uncaria gambir* leaves and *Macadamia* nut shells [115,117].

Chemically synthesized and non-functionalized AuNPs exhibit greater activity against Gram-negative than Gram-positive bacteria [93]. This relationship was also observed for the majority of AuNPs biosynthesized using plants [111,114,115,117]. These were AuNPs obtained using extracts from *Uncaria gambir* leaf, *Platycodon grandiflorus* flowers, *Macadamia* nut shells and *Annona muricata* leaves. Sometimes, biosynthesized AuNPs exhibited better activity against Gram-positive bacteria. For example, AuNPs synthesized using *Aloe vera* extract, *Citrullus lanatus* and *Catharanthus roseus* leaves demonstrated antibacterial activity against *S. aureus*, *S. epidermidis*, and *S. pyogenes*, and *S. aureus*, respectively [118,123,124]. However, in most cases, biosynthesized AuNPs exhibit antibacterial activity against both types of bacteria. For example, AuNPs obtained using *Ananas comosus* fruit extract were effective against Gram-positive and Gram-negative bacteria inhabiting the aquatic environment. The obtained effect was attributed to the supporting effect of bromelain present in the extract [125]. The biosynthesis of AuNPs using *Garcinia indica* and *Garcinia cambogia* fruit extract was also carried out which resulted in small, spherical AuNPs with sufficient antibacterial activity against the Gram-positive bacteria *B. subtilis* and the Gram-negative *E. coli* [126]. Additionally, AuNPs biosynthesized using the leaf extract of *Allium ampeloprasum* showed sufficient activity against Gram-positive (*S. aureus*, *B. subtilis*) and Gram-negative (*E. coli, P. aeruginosa*) bacteria [113].

Some AuNPs synthesized using plant extracts do not have antibacterial properties but exhibit a different kind of biological activity. For example, AuNPs obtained using the ethanolic extract of *Moringa oleifera* or the aqueous extract from *Dracocephalum kotschyi* leaves showed no antibacterial activity. However, the ethanolic extract of *Moringa oleifera* causes antiepileptic activity and the aqueous extract from *Dracocephalum kotschyi* leaves is cytotoxic to HeLa and K562 cell lines [127,128].

### 3.1. Role of AuNPs Shape and Size

Controlled synthesis of NPs is aimed at tailoring AuNPs to the appropriate shape and size for the applicable bacterial cell system [24]. Moreover, a significant relationship has been established between the size, shape and concentration of the obtained AuNPs and their antibacterial properties [58]. The small size of NPs allows them to penetrate into the cell and influence the various cellular processes [25,129]. For example, 6 nm AuNPs showed less toxicity against *B. subtilis*, whereas 2 nm AuNPs were able to lyse the bacteria [130]. Very small AuNPs can interact with the bacterial surface and penetrate bacteria cells despite the thickness of their cell walls [52,131,132]. Small AuNPs biosynthesized using the leaf extract of *Uncaria gambir* and triethanolamine blocker exhibited antibacterial activity against Gram-positive and Gram-negative bacteria which was dependent not on the concentration of AuNPs but on their size [115]. However, an opposite trend regarding antibacterial activity can be observed in the case of biosynthesized AuNPs with a different type and composition of envelope coating. The effect of AuNP size on antibacterial activity was investigated using AuNPs biosynthesized using dextrose isolated from plants. Optimal, average, and no antibacterial activity was found against *E. coli* using 120 nm, 60 nm, and 20 nm AuNPs, respectively [132].

Often, there is no clear dependence on the concentration and size on the antibacterial activity of AuNPs biosynthesized from plants. In such cases, it seems that the polymorphism of NPs may play a role. AuNPs synthesized from *Macadamia* nut shells exhibited no relationship between AuNP concentration and size on antibacterial activity, although the size distribution of the obtained AuNPs was quite large (50–200 nm) [117]. Another characteristic feature of the obtained AuNPs was their different shapes. Spherical, hexagonal and triangular AuNPs can be formed via biosynthesis. Thus, the observed differences in antimicrobial activity could be related to the degree of polymorphism of the obtained AuNPs. Whether spherical, hexagonal or triangular AuNPs will form is dependent on the physicochemical parameters (i.e., the *v*/*v* ratio of the reactants, HAuCl_4_ solution concentration, temperature, pH and the duration of the reaction) of the synthesis reaction [18,35,80]. However, for AuNPs biosynthesized and biofunctionalized by bioactive molecules from plant extracts, the shape of the NPs does not have a large significance on the NPs’ biological activity as compared to naked AuNPs or other types of NPs [133,134,135].

However, the size, surface charge, and the antibacterial activity of AuNPs depends on the specific surface area (i.e., the ratio of the surface area to the volume of the NP) [136]. As the specific surface area of an AuNP increases, more biomolecules may be attached and the NP has a larger surface area to interact with the bacterial cell [14,137]. Similarly, the spherical and elongated shape of AuNPs may facilitate their passage through cell membranes, as found in studies investigating the effects of AuNP size and shape on the efficiency of cellular uptake [136,138]. Hence, tailoring the surface-area-to-volume ratio of AuNPs is one of the most favorable parameters to adjust in order to influence the way NPs interact with bacterial cells [133,134,135].

### 3.2. Role of AuNP Envelope

Comparative studies were carried out to test the antibacterial activity of chemically and biologically synthesized AuNPs. AuNPs which do not possess an envelope comprised of bioactive molecules exhibited no antibacterial activity. On the other hand, biosynthesized AuNPs demonstrated a large degree of antimicrobial activity [139]. The results of the performed antibacterial tests suggest that naked AuNPs had no effect on bacteria growth or their vitality, whereas AuNPs possessing an envelope formed via bioactive compounds during biosynthesis inhibited the growth of bacterial cells [140]. In addition, AuNPs synthesized using *Medinilla speciosa* (a plant known for its antibacterial properties) infusion enhanced the antibacterial effects compared to the extract alone [36]. This approach is less frequent, i.e., when plant extracts with antibacterial properties are used for the synthesis of AuNPs (Table 3).

However, regardless of whether the extract contains phytochemicals with antibacterial properties or not, the most common antibacterial tests for the extract itself are negative. This is because such antibacterial tests are carried out using plant extract concentrations that are the same as those used for the synthesis of AuNPs. Extracts for the synthesis of NPs are diluted because a very low concentration of phytochemicals is sufficient to carry out the reaction and spectrophotometric methods can characterize the obtained AuNPs at these concentrations [27,78].

The special properties of plant extracts cause the formation of the envelope, which also acts as a stabilizing layer preventing the aggregation of AuNPs and the formation of larger structures [39]. The zeta potential measures the surface charge of NPs and is used to assess AuNP stability. Zeta potential values vary with pH, and therefore they can be modified by changing the concentration of hydrogen ions. The greater the absolute value of the zeta potential, the more stable the AuNPs are. Mutual repulsion of neighboring molecules prevents their aggregation [3]. The lowest zeta potential values are observed in a strongly acidic pH. For example, AuNPs obtained using *Punica granatum* fruit extract exhibited small, negative zeta potential values, indicating they were less stable and therefore had a greater tendency to aggregate and form large AuNPs [18]. On the other hand, AuNPs biosynthesized using *Ananas comosus* extract were stable for 30 days. High stability was a result of the potential barrier created by the interaction between the weak Van der Waals bonds and the repulsive forces of the electrostatic interaction [125]. The surface charge of the NP depends on the envelope composition (i.e., the charge of the biomolecules that form the envelope) and the ionic composition of the extract. Due to the negative charge of the bacterial cell wall, the positive surface charge of AuNPs may facilitate greater interaction with bacterial cells than neutral or negatively charged AuNPs [145]. Hydrophobic AuNPs have a positive surface charge. As a result, hydrophobic AuNPs can create spatial aggregates on the surface of bacterial cells [130]. Similarly, small hydrophobic AuNPs can more easily penetrate lipid membranes and enter the cell [133,146]. The mode of interaction with bacterial cells also depends on the bacterial strain [130]. Many studies indicate that the antibacterial activity of AuNPs biosynthesized using plant extracts is strongly related to the composition of the envelope [147,148,149].

### 3.3. Composition of AuNP Envelope

The ability of plant extracts to stabilize AuNPs plays an important role. The biomolecules responsible for the reduction of gold ions are likely also responsible for the stabilization of AuNPs [150]. The phytochemicals present on the surface of AuNPs make them stable colloids [106]. For example, the flavonoids present in the *Trigonella foenum-graecum* extract were responsible for both the reduction of gold ions and the stabilization of AuNPs due to the presence of the carboxyl group [151]. Hence, studies investigating the characterization and composition of plant extracts used for the bioreduction of Au^3+^ ions are a key issue [150].

With regard to antibacterial activity, AuNPs possessing an envelope formed via bioactive molecules showed greater effectiveness than naked AuNPs [140]. Many studies show that the group of compounds most often responsible for the reduction of gold ions and the stabilization of AuNPs are flavonoids [38,39,41]. There are about 1000 variants of flavonoid derivatives, and these compounds act as pigments to protect plants against radiation [54,55]. The number of hydroxyl groups in the flavonoid compound, their location, and the flavonoid’s degree of oxidation are the most important structural properties related to AuNP synthesis and stabilization. Moreover, the presence of hydroxyl groups has great importance in the reduction of gold ions and the stabilization of AuNPs [54,55]. Flavonoids are also known to form chelate bonds with metals, thus trapping metals and inactivating certain enzymes. The affinity of flavonoids for AuNPs can also be explained by their ability to chelate metals [152]. Flavonoids possess many types of biological activity that can be used in biomedicine, such as the ability to strengthen blood vessel walls (rutin, hesperidin, diosmin), stimulate the production of anti-inflammatory prostaglandins, scavenge free radicals and improve blood circulation in vessels. At the same time, the antibacterial, antiviral and antifungal properties of flavonoids are being reported more frequently [153]. For example, the analysis of FTIR spectra of AuNPs synthesized with the extracts of *Terminalia arjuna*, *Polygonum fagopyrum*, *Couroupita guianensis*, *Solanum indicum*, *Malus domestica*, *Citrullus lanatus*, *Cornus mas* and grapes revealed that mainly flavonoids and phenolic compounds were forming a stabilizing envelope around the AuNPs [9]. Furthermore, the envelope surrounding AuNPs synthesized using extract from *Imperata cylindrica* leaves consisted mainly of phenolic compounds [154]. The stabilizing envelope of AuNPs synthesized using *Medinilla speciosa* infusion consisted of polyphenolic compounds. Naringin and quercetin present in the extract were specifically responsible for the antibacterial effect [36]. The envelope surrounding AuNPs synthesized using the pod extract of *Gymnocladus assamicus* consisted mainly of phenolic acids such as gallic and protocatechuic acids and kaempferol. In the case of AuNPs synthesis using *Muntingia calabura* leaf extract, the envelope was not only composed of flavonoids, but also tannins and saponins [155].

Another group of compounds that has a significant impact on the stability of AuNPs are fatty acids. In the research conducted by Abdel-Raouf et al. (2017), 22 components, including fatty acids such as palmitic acid, oleic acid, and stearic acid, formed AuNPs envelopes [55]. Palmitic acid is a very strong antiseptic, and its presence increased the antibacterial properties of AuNPs. Similarly, palmitic acid also stabilized the AuNPs and thus prevented their aggregation [55]. Additionally, secondary metabolites such as terpenoids, flavonoids and aliphatic amines, which form a stabilizing envelope around AuNPs, were detected in the *Salix alba* extract. For this reason, such compounds are isolated and used for the synthesis of AuNPs. For example, terpenoids included in plant extracts can act against *S. aureus* bacteria and change the permeability of the membrane [156].

Proteins are a frequent component of AuNP envelopes synthesized using plant extracts and likely responsible for the reduction of gold ions and stabilization of AuNPs. Moreover, the presence of proteins in the AuNP envelope may also determine the type of interaction with the bacterial cell wall [157]. In the case of AuNPs synthesized using *Annona muricata* leaf extract and extract from stem of *Periploca aphylla*, the envelope consisted of flavonoids, terpenoids and proteins. The envelopes of AuNPs obtained using the leaf extracts of *Allium ampeloprasum* and *Eclipta prostrata* were composed of phenolic compounds and proteins [88,113,114,157]. The presence of proteins, alkaloids and flavones was also found in the envelope of AuNPs synthesized using *Nigella arvensis* leaf extract [1].

Studies investigating the composition of the AuNP envelope indicate that the presence of bioactive secondary metabolites may play an important role in the reduction of gold ions and the stabilization of AuNPs. In addition, studies have confirmed the participation of amine and amide functional groups, as well as hydroxyl and aromatic groups, in the reduction of gold ions and the role of these groups in stabilizing AuNPs [55].

### 3.4. Antibacterial Properties of Plant Extracts and Isolated Phytocompounds

Most often, AuNPs are synthesized from extracts without antibacterial properties, or such activity of the extracts is not tested because these studies aim to characterize the reduction ability of extracts and the characteristics of the obtained AuNPs. Interestingly, AuNPs may exhibit antibacterial activity even if the extract used during the biosynthesis did not previously exhibit antibacterial activity on its own. Furthermore, several examples of AuNPs synthesized using extracts or phytochemicals to achieve antibacterial activity demonstrated that the presence of AuNPs always enhanced the antibacterial action. Therefore, future studies should first test the antibacterial activity of the extract itself or the phytochemicals isolated from it, and then assess the ability of the extract or extract components to reduce gold ions.

Interestingly, many extracts and extract components have already been tested in this manner (Table 4). Extracts from plants used in traditional medicine, generally known as herbs, are often used for the synthesis of AuNPs [158].

The antibacterial activity of extracts obtained from *Achillea millefolium*, *Caryophyllus aromaticus*, *Melissa officinalis*, *Ocimum basilicum*, *Psidium guajava*, *Punica granatum*, *Rosmarinus officinalis*, *Salvia officinalis*, *Syzygium jambolanum* and *Thymus vulgaris* plants was investigated using 14 strains of bacteria. Extracts from *Thymus vulgaris*, *Rosmarinus officinalis*, *Syzygium jambolanum*, *Punica granatum*, *Psidium guajava* and *Ocimum basilicum* exhibited antibacterial activity against *P. aeruginosa*, *C. albicans*, *Proteus* sp., *B. subtilis*, *S. aureus*, *K. pneumoniae* and *E. aerogenes* [158]. Antimicrobial activity of substances isolated from plants, such as eugenol, benzoyl acid and cinnamic acid, was observed against *S. aureus*, *S. choleraesuis*, *B. subtilis*, *C. albicans*, *K. pneumoniae*, *E. aerogenes* and *E. coli* [72,158]. The composition and antimicrobial activity of leaf extracts from *Moringa oleifera*, *Magnolia acuminata*, *Prunus cerasus* and *Leucaena leucocephala* were also investigated. Positive antibacterial test results were observed against *E. coli*, *S. aureus* and *B. subtilis*. Mainly, extracts contain alkaloids, saponins, tannins, phenolic acids, flavonoids, steroids and glycosides [163]. Out of the extracts derived from *Digitalis purpurea*, *Sanicula europaea*, *Anthemis pungens*, *Ecballium elaterium*, *Urtica dioica*, *Nerium oleander*, *Tilia argentea*, *Juglans regia*, *Pistacia* sp. and cardiac glycoside isolated from *Digitalis purpurea*, the extract from *Tilia argentea* showed antibacterial activity against the greatest number of bacterial strains (i.e., *E. coli*, *B. subtilis*, *K. pneumoniae*, *S. aureus* and *Aeromonas* sp.). On the other hand, extracts from *Anthemis pungens* exhibited antibacterial activity against two strains of *E. coli* and *S. aureus*, whereas *Pistacia* sp. exhibited activity against *E. coli* and *B. subtilis* [120].

In addition, the synthesis of AuNPs using ethanol extracts usually provides greater antibacterial efficacy compared to aqueous extracts [55]. In the case of methanol extracts from *Allium sativum*, *Caryophyllus aromaticus* flowers, rhizomes of *Zingiber officinale* and *Psidium guajava*, *Cymbopogon citratus* and *Mikania glomerata* leaves, all of the extracts exhibited antibacterial activity against *E. coli*, *Salmonella*, *S. aureus* and *Enterococcus* sp. However, different MIC_50%_ and MIC_90%_ values were obtained for the individual extracts [166]. Similarly, a zone of inhibition of bacterial growth was observed for methanol extracts from the stems, leaves and roots of *Sclerocarya birrea* and *Sterculia setigera* against *E. coli*, *C. albicans*, *S. aureus* and *A. niger* [167]. Additionally, the metal extract from the leaves of *Premna pubescens* showed antibacterial activity against *S. aureus* [168].

On the other hand, results obtained using aqueous extracts should be considered separately. The antimicrobial activity of alcohol extracts is usually much greater and affects a greater number of bacterial strains than aqueous extracts, which can be attributed to the antiseptic properties of alcohols. Thus, the size of the bacterial growth inhibition zone is not only a result of the properties of the phytochemicals contained in the extract. This relationship is most evident in comparative tests carried out using aqueous, alcoholic and other extracts from the same plants. Such studies were carried out using extracts from the tubers of *Asparagus falcatus*, the whole plants of *Asteracantha longifolia* and *Epaltes divaricata*, the roots of *Vetiveria zizanioides*, and the seeds of *Coriandrum sativum*. Only one aqueous extract (i.e., *Epaltes divaricata*) achieved a positive antibacterial result, whereas the ethanolic and hexane extracts resulted in eight positive antibacterial tests [121]. All types of extracts were tested against one bacterial strain of *S. aureus*. The antimicrobial activity of the extracts from leaves, branches, and flowers of *Luma apiculata* obtained in various organic solvents, including ethanol, methanol, hexane and distilled water, was studied. Only the hexane extracts were effective against *S. aureus*, *S. epidermidis*, *S. saprophyticus*, *Enterococcus* sp., *A. baumannii*, *P. aeruginosa* and *E. coli* [161]. On the other hand, both ethanolic and aqueous extracts from leaves of *Arum maculatum* exhibited antibacterial activity against all of the investigated bacterial strains including *E. coli*, *S. aureus*, *L. monocytogenes*, *S. enteritidis* and *P. aeruginosa*. The results differed only slightly in MIC_50%_ values for both types of extracts [160]. Studies were also carried out comparing the antimicrobial activity of methanol extracts from different parts of the same plant [122]. All extracts derived from the flowers, leaves and seeds of *Cleome coluteoides* showed antibacterial activity against *B. cereus* and *S. aureus*. Interestingly, only slight differences in the size of the zone of inhibition were observed, but the same relationship was evident for both bacteria. The leaf extract was the most effective, followed by the flower extract, and the smallest zone of inhibition was found for the seed extract [122]. Similar studies were carried out for methanol extracts obtained from the leaves, roots, and seeds of *Crotalaria bernieri* against 17 strains of bacteria. The leaf extract was most effective and demonstrated antibacterial activity against *E. aerogenes*, *P. aeruginosa*, *V. parahaemolyticus*, and *P. mirabilis*. Conversely, the root extract exhibited only two positive antibacterial activity results against *S. enteritidis* and *P. mirabilis*. The seed extract was the least effective, for which only one positive result was observed for *Y. enterocolitica* [159].

## 4. Mechanism of the Antibacterial Activity of AuNPs Synthesized from Plants

The antibacterial properties of biosynthesized AuNPs differ based on their size, shape, concentration, composition, envelope composition, stability, and surface charge. The mechanism of cellular toxicity is important. However, because the antibacterial effects of biosynthesized AuNPs is dependent on so many parameters, identifying the root of cellular toxicity remains difficult [25].

**Figure 3:**
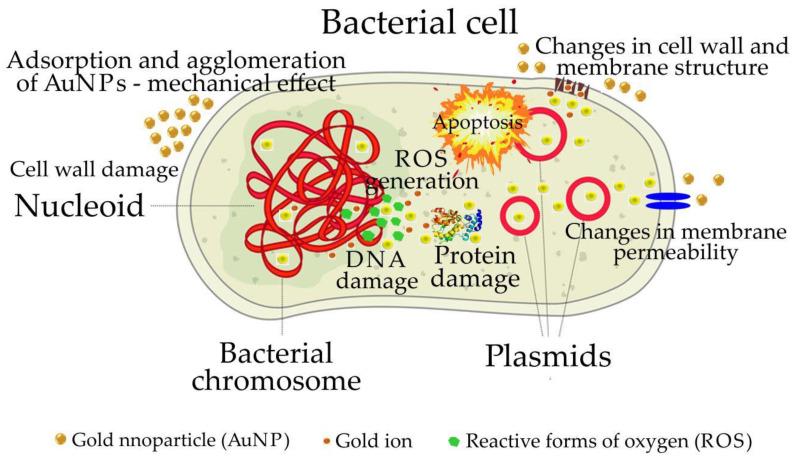
Mechanism of antibacterial action of AuNPs biosynthesized from plants and unreduced gold ions.

The mechanisms responsible for the antibacterial activity of AuNPs are oxidative stress, the release of gold ions and non-oxidative stress (Figure 3) [3,28]. The antibacterial properties of biosynthesized AuNPs are also determined by the presence of secondary metabolites derived from plant extracts. In one study, the biological activity of AuNPs, including antibacterial properties, was determined by the concentration of tannins, flavonoids, phenols and aromatic compounds present in their envelopes [169]. For example, proteins and enzymes were present in the AuNPs obtained using the *Nigella arvensis* leaf extract, and they increased the antibacterial activity of AuNPs [1]. As a result, the AuNPs were able to interact with the bacterial cell wall, change its structure and eventually destroy it [28]. Thus, the first interaction of AuNPs with bacteria is at the molecular level with the cell wall. Therefore, the envelope composition of AuNPs has a great impact on the antibacterial activity of the NPs and determines the stability and surface charge of AuNPs.

### 4.1. Damage to the Cell Wall

The cell wall of bacteria aids in maintaining cell shape and is the first basic barrier which protects the cell against mechanical and osmotic damage. The antibacterial activity of bacteria depends on the bacterial strain (i.e., the structure of the cell wall) [28,35,93,170]. The surface interactions between AuNPs stabilized by phytochemicals and the bacterial cell wall are also important [125]. AuNPs aggregating on the cell surface can disturb the permeability of the cell membrane by causing pits, fissures and pores [69,133,146,171]. The concentration of adherent AuNPs will depend on the NP surface charge. AuNPs accumulating on the cell surface affect the cell wall by disturbing the equilibrium state and changing the Gibbs energy in accordance with the Le Chatelier concept [15]. Large, hydrophilic biosynthesized AuNPs cannot penetrate the lipid membrane [93,133,146]. On the other hand, small AuNPs with hydrophobic surfaces can freely pass through the pores in the lipid membranes which facilitates their internalization inside the cell [130,133,136,138]. However, the appropriate concentration of adherent AuNPs and specific interaction are necessary for the internalization of AuNPs [146]. The hydrophilic or hydrophobic properties and the surface charge of the NPs depend on the stabilizing compounds within the extract [93,133,146]. When biosynthesized AuNPs penetrate inside bacteria, they can lead to cell death by disrupting bacterial metabolism by interacting with the mitochondria and other organelles and by intercalating with bacterial DNA [1,15,125].

At the molecular level, damage to the cell wall may consist of non-specific binding of small AuNPs adhering to the surface of bacteria with transpeptidase, which leads to increased membrane permeability, cell lysis and DNA leakage [7,172]. Additionally, the accumulation of large AuNPs on the cell surface because of the attractive electrostatic interaction forces may increase the permeability of the membrane. The critical concentration of adhered AuNPs may lead to morphological changes in the cell wall and/or increase its permeability, which in turn causes cell death [7,35]. The first effect of the alterations in membrane permeability is the disruption of the membrane ion transport selectivity which may initiate depolarization of the lipid membrane. Then, an increased influx of Ca^2+^ ions into the cell is observed. The increase in the concentration of calcium ions in the cytoplasm initiates cell death in an apoptosis-like manner [7,109,173,174,175]. The interaction between biosynthesized AuNPs and negatively charged lipopolysaccharides (LPS) can also affect the membrane permeability of Gram-negative bacteria because LPS loses stability at a critical concentration of AuNPs. Such a mechanism was observed in the case of AuNPs biosynthesized using *Abutilon indicum* leaf extract [35,176]. On the other hand, the critical concentration of adhered AuNPs, particularly large AuNPs which cannot penetrate cell membranes, strongly influences deformations in the cell shape [7,177,178,179]. Bacterial cell deformation occurs as a result of the mechanical action of AuNPs on the membrane, leading to cell rupture and death. This physical mechanism of action on bacteria cell death has been observed using perfectly spherical chemically synthesized AuNPs and biosynthesized AuNPs from dextrose [132,180]. Mechanical damage of the membrane also leads to cell lysis, resulting in the leakage of cytoplasm and nucleic acids [132].

### 4.2. Damage of Proteins and DNA

Proteins fulfill important structural and catalytic functions in all living organisms [28]. Membrane proteins and intracellular components influence cell division, respiration, and ultimately cell survival. AuNPs showing affinity to compounds containing nitrogen and sulphur atoms can alter or break the structure of proteins by bonding to their thiol and amino groups [5,7,73,107,176,181,182,183,184]. For example, the mechanism of antibacterial action of AuNPs synthesized using the leaf extract from Nigella arvensis was due to the AuNPs binding to the external components of the bacterial cell wall, which caused changes in the wall structure enabling AuNP penetration into the cell to disrupt cellular respiration [1,7]. Inside the cell, AuNPs interact with DNA and block transcription which inhibits cell growth and senescence, resulting in cell degradation and bacteria death [1].

Moreover, AuNPs can prevent the binding of the ribosomal subunit to tRNA and also disrupt the membrane potential by inhibiting ATPase activity. Inhibiting ATPase activity reduces the level of ATP and stimulates the formation of reactive oxygen species (ROS), which affects other cellular structures [105,185,186]. ROS generated by AuNPs inhibit respiratory enzymes and cause an increase in oxidative stress, which in turn leads to cell death [35,52]. The ability of AuNPs to generate ROS was confirmed in an experiment in which bacterial cells were treated with AuNPs while adding 2,7-dichlorodihydro- fluorescein diacetate (DCFH-DA). DCFH-DA is a pigment that emits green fluorescence only in the presence of ROS while it is oxidized. In cells treated with AuNPs and DCFH-DA, fluorescence was observed. Conversely, no fluorescence was observed in cells treated with DCFH-DA alone [187]. The mechanism of action related to ROS generation was found in AuNPs synthesized using *Ocimum tenuiflorum* flower and leaf extract, *Azadirachta indica* and *Mentha spicata* leaves, and peel extract from *Citrus sinensis* [15]. Consequently, the ROS generated by AuNPs can affect metabolic replication, transcription and cell division, since ROS can cause DNA mutations. These modifications can lead to saccharide fragmentation and/or double helix breakage [7,28].

Therefore, two steps occur after AuNPs enter the cell: reduction of the produced ATP, which results in a reduction of metabolic activity, and the disturbance of ribosome binding, which leads to inhibition of protein synthesis [7,45,93,188,189].

### 4.3. Changes in the Expression of Metabolic Genes

AuNPs generate not only singlet oxygen but also other radicals. ROS are harmful to cells because they react with amino acids such as methionine, vitamins including beta-carotene, unsaturated fatty acids, proteins and steroids [89,190]. Enzymatic antioxidant cell defense systems in bacterial cells, e.g., superoxide dismutase (SOD) and glutathione peroxidase (GPx), are regulated by ROS-dependent signals [191]. The level of deoxidants varies depending on the activity of the respective genes in order to inhibit oxidative damage. Microbes can change metabolic pathways to those that are able to repair damaged cellular structures using ROS, such as the cell membrane or DNA [28]. Oxidative stress is a normal cellular process that occurs in several phases of cell signalling. However, if the balance between ROS production and the biological ability to deoxidise ROS species becomes too great, the effects can rapidly become very harmful [45]. Under normal conditions, the production and removal of ROS is counterbalanced by appropriate enzyme systems. If ROS levels are too high, the redox syndrome can lead to cell death by damaging basic structures, including metabolic pathways and DNA [28].

Other studies indicate that the redox syndrome does not have to be associated with an increase in ROS concentration. It may be related to the direct damage of the GPx enzyme by AuNPs, which contributes to oxidative cell damage [36,190,191,192,193]. Such a mechanism was found in the case of *E. coli*. The oxidative stress caused by AuNPs was associated with a decrease in GPx concentration as a result of the direct destructive action of AuNPs. The increase in ROS concentration was not found to cause the oxidative stress. This oxidative imbalance induced apoptosis in a similar fashion as the ROS-independent apoptosis of mammalian cells [192].

## 5. Conclusions

The biological synthesis of AuNPs has gained increased interest due to the simple, cheap and eco-friendly procedure, as well as the wide availability of biological material to carry out the syntheses. Moreover, AuNP biosynthesis using plant extracts is a particularly promising solution due to the vast number of applications in biomedicine.

In addition to their unique physicochemical properties, AuNPs biosynthesized using plant extracts or bioactive compounds isolated from plants have specific biological properties and are biocompatible and non-toxic. Biosynthesized AuNPs can have different shapes, sizes, surface potentials and stabilities, and the biomolecules present within the AuNP envelope and on the AuNP surface depend on the composition of the plant extract. The structure and envelope composition of AuNPs formed during biosynthesis depends on the physicochemical conditions of the reaction and type of extract used. The biological properties of AuNPs depend on the morphological and surface properties of the NPs and the type and composition of the envelope formed during biosynthesis.

One of the biological activities exhibited by biosynthesized AuNPs is antibacterial action, which is particularly interesting and desired in the era of increasing bacterial resistance for which new antibacterial agents are sought after. The mechanism of antibacterial action of biosynthesized AuNPs on bacteria is very complex, and NPs of different morphology and envelopes may exhibit differences in antibacterial activity because in each case a different path of cell death is triggered. Interestingly, the extract itself may not contain antibacterial substances, just as AuNPs with a given morphology may not exhibit antibacterial activity. However, AuNPs of a given morphology which did not previously exhibit antibacterial activity may become active against bacteria using a specific extract as a result of biosynthesis. Additionally, the level of antimicrobial activity depends on the bacterial strain and the concentration of AuNPs. Although some research observed antimicrobial activity independent of the concentration of AuNPs, this is the exception rather than the rule. Furthermore, the level of antimicrobial activity of biosynthesized AuNPs may be comparable to that of standard antibiotics and even higher.

The composition of the plant extract used for synthesis is of particular importance because the initiation of the antibacterial action depends on the adsorption of NPs to the surface of the bacterial cell. This is possible due to the appropriate surface charge of the AuNP and the interaction at the molecular level with the components of the bacterial cell wall. Both the surface charge of the biosynthesized AuNPs and the composition of the biomolecules in the envelope depend on the type of extract used. Additionally, if the extract itself possesses antibacterial properties, AuNPs synthesized using the extract always enhance the antibacterial activity. Due to the promising results, the demonstrated activity against many bacterial strains and the increasingly well-known mechanism of action of biosynthesized NPs, these AuNPs may constitute new antimicrobial agents that can be used alone or in combination with antibiotics as they have great potential.

The unique physicochemical and biological properties of AuNPs, such as small size, surface charge, SPR effect, stability, envelope biomolecules, and biocompatibility predispose them to various biomedical applications. Biosynthesized AuNPs have been considered as the most promising nanomaterial in target delivery, controlled drug release, antimicrobial drugs, biosensors, hyperthermia, imaging, and theranostics.

## Figures and Tables

**Table 2:** Antibacterial activity and mechanism of antibacterial action of AuNPs synthesized from plant extracts.

Plant	Kind of Extract	Reducing and Stabilizing Agent	Shape and Size of AuNPs	Bacterial Strains (MIC [μg/mL])	Mechanism of Antibacterial Activity	Another Activity	References
*Ocimum tenuiflorum*	Flower and leaf; water extract	Phenolic compounds	Spherical; 20–25 nm	*S. aureus*, *P. aeruginosa*, *K. pneumoniae* (512–600)	Disturbing the membrane permeability and cell metabolism	-	[15]
*Azadiriachta indica*	Leaf; water extract						
*Mentha spicata*	Leaf; water extract						
*Citrus sinensis*	Peel; water extract		Spherical; 25–30 nm				
*Nigella arvensis*	Leaf; water extract	Flavones, proteins	Spherical, triangular, pentagonal, hexagonal; 3–37 nm	*E. coli*, *B. subtilis*, *S. aureus* (62.5–125)	Cell wall structure and cell respiration disruption	Antioxidant, cytotoxic, catalytic capacity	[1]
*Thymus vulgaris*	Whole plant	Unknown	Spherical; 6–35 nm	*B. subtilis* (15.62)	Unknown	Antioxidant; non-toxic	[98]
*Cryptolepis buchanani*	Leaf; water extract	Phenolic compounds	Spherical; 11.1 nm	*S. aureus*, *A. baumannii* (0.209)	Unknown	Catalytic capacity	[99]
*Cola acuminata*	Fruits; water pulp	Flavonoids, terpenoids	Spherical; 14–22 nm	*S. aureus*, *E. coli*, *P. aeruginosa*, *B. cereus*	Unknown	-	[100]
*Olea europaea* *Acacia nilotica*	Fruit and husk; mixture of water extracts	Phenolic compounds, terpenoids	Spherical; 45 nm	*E. coli*, *Bacillus* sp., *P. aeruginosa*, *Klebsiella* sp.	Unknown	Cytotoxic	[101]
*Pistacia atlantica*	Leaf and fruit, water extract	Unknown	Spherical; 50–60 nm	*S. aureus*, *E. coli*	Unknown	Antioxidant, cytotoxic	[98]
*Amomum villosum*	Fruit; water extract	Phenolic compounds, tannins, terpenoids	Spherical; 10–15 nm	*S. aureus*, *E. coli*	Unknown	Antioxidant, cytotoxic, catalytic capacity	[102]
*Cannabis sativa*	Stem; water extract	Cannabinoids, terpenoids, flavonoids, phenolic compounds	Spherical, triangular, elongated, hexagonal; 12–18 nm	*S. epidermidis*, *P. aeruginosa*, *E. coli* (25–50)	Unknown	-	[9]
*Citrullus lanatus*	Fruit; water extract	Citrulline, proteins, carotenoids	Spherical; 20–140 nm	*B. cereus*, *S. aureus*, *S. typhimurium*	Unknown	-	[103]
*Medinilla speciosa*	Fruit; water infusion	Phenolic compounds, amino acids	Spherical; 200–450 nm	*P. aeruginosa*, *S. aureus*	Oxidative stress	-	[36]
*Cynodon dactylon*	Whole plant; water extract	Unknown	Spherical; 200–450 nm	*E. cloacae*, *B. cereus*, *S. haemolyticus*, *S. petrasii*	Oxidative stress	Cytotoxic	[104]
*Areca catechu*	Nuts; water extract	Flavonoids, proteins	Spherical; 13.7 nm	*E. coli*, *S. aureus*, *K. pneumonia*, *P. aeruginosa*	Unknown	Antioxidant, catalytic capacity, cytotoxic	[77]
*Petroselinum crispum*	Leaf; water extract	Unknown	Spherical, semi-rod, flower-shaped; 17–50 nm	*Enterobacter ludwigii*	Unknown	-	[105]
*Salix alba*	Leaf; water extract	Amides, proteins	Spherical; 50–80 nm	*S. aureus*	Unknown	Antifungal	[106]
*Jasminum auriculatum*	Leaf; water extract	Unknown	Spherical; 8–37 nm	*S. pyogenes*, *S. aureus*, *E. coli*, *K. pneumonia*	Unknown	Catalytic capacity, antifungal, cytotoxic	[107]
*Solanum nigrum*	Leaf; water extract	Unknown	Spherical; 50 nm	*S. saprophyticus*, *B. subtilis*, *E. coli*, *P. aeruginosa*	Unknown	Antioxidant	[108]
*Aloysia triphylla*	Leaf; water extract	Flavonoids	Spherical; 40–60 nm	*E. coli*, *S. aureus* (50)	Unknown	Catalytic capacity	[109]
*Plumeria alba*	Flower; water extract	Unknown	Spherical; 28, 3.4 nm	*E. coli* (400)	Unknown	Catalytic capacity	[110]
*Platycodon grandiflorum*	Leaf; water extract	Flavonoids	Spherical; 15 nm	*E. coli*, *B. subtilis* (10)	Unknown	-	[111]
*Peganum harmala*	Leaf; water extract	Polyphenols, alcohols	Spherical; 43.44 nm	*E. coli*, *S. aureus*	Unknown	-	[112]
*Allium ampeloprasum*	Leaf; water extract	Phenolic, aromatic compounds, proteins	Spherical; 22.76 nm	*S. aureus*, *B. subtilis*, *E. coli*, *P. aeruginosa*	Cell wall, mitochondrial and ribosome damage	Antifungal	[113]
*Annona muricata*	Leaf; water extract	Flavonoids, terpenoids, proteins	Spherical; 25.5 nm	*C. sporogenes*, *S. aureus*, *E. faecalis*, *K. pneumonia*	Unknown	Antifungal	[114]
*Uncaria gambir*	Leaf; water extract	Unknown	Spherical, triangular, hexagonal; 32.52 nm	*E. coli*, *S. aureus* (50)	Cell wall structure disruption	-	[115]
*Pergularia daemia*	Leaf; water extract	Unknown	Spherical; 3–15 nm	*E. coli*, *B. subtilis*, *P. aeruginosa* (300)	Membrane permeability and cell respiration disruption	-	[116]
*Macadamia*	Nut shell; water extract	Unknown	Spherical, triangular, hexagonal; 50–200 nm	*E. coli*, *S. epidermidis*	Unknown	-	[117]
*Aloe vera*	Leaf; water extract	Alcohols, phenolic compounds	Spherical; <15 nm	*E. coli*, *S. aureus* (10)	Unknown	-	[118]
*Azadirachta indica*, *Zingiber officinale*	Leaf; mixed water extracts	Unknown	Spherical, triangular, hexagonal; 32.52 nm	*S. mutans*, *S. aureus*, *E. faecalis*	Unknown	-	[119]
*Tilia argentea*	Leaf; water: PBS extract	Unknown	Unknown	*E. coli*, *B. subtilis*, *K. pneumoniae*, *Aeromonas* sp., *S. aureus* (250)	Unknown	-	[120]
*Hibiscus sabelariffa*	Flower; water and ethanol extracts	Unknown	Unknown	*E. coli*, *S. aureus*, *P. aeruginosa*, *V. parahaemolyticus*, *S. enteritidis*, *B. cereus*	Changes in internal pH of bacterial cell	-	[72]
*Syzygium* *aromaticum*	Flower; water and ethanol extracts						
*Rosmarinus* *officinalis*	Leaf; water and ethanol extracts						
*Epaltes divaricata*	Whole plant; ethanol extract	Unknown	Unknown	*S. aureus*	Unknown	-	[121]
	Whole plant; hexane extract						
	Whole plant; water extract						
*Vetiveria* *zizanioides*	Root; ethanol extract	Unknown	Unknown	*S. aureus*	Unknown	-	[121]
	Root; hexane extract						
*Cleome coluteoides*	Stem; methanol extract	Polyphenols	Unknown	*B. cereus*, *S. aureus*	Unknown	-	[122]
	Flower; dichloromethane, methanol, ethyl acetate extracts						
	Leaf; dichloromethane, methanol, ethyl acetate extracts						

**Table 3:** Examples of plant compounds used in the synthesis of AuNPs.

Compound/Kind of Solution	Plant	Shape and Size of AuNPs	Bacterial Strains (MIC [μg/mL])	Mechanism of Antibacterial Activity	Other Activities	Function	References
Pectin; water solution	*Musa paradisiaca*	Spherical; 8 nm	-	-	Cytotoxic	Anticancer drug	[64,65,68,69] [141,142]
	Orange peels	Spherical; 7–13 nm			Anti- inflammatory	Drug carrier	
Flavonoid, tricetin	pollen of *Myrtaceae*	Spherical; 12 nm	*B. licheniformis*, *S. aureus*, *A. pittii*, *E. xiangfangensis*, *E. fergusonii*, *P. mirabilis*, *P. aeruginosa*, *A. enteropelogenes*	Unknown	Biocompatibility	Chitozan carrier, antibacterial drug	[143]
Polyphenol, epigallocatechin 3-gallate; water solution	Green tea	Spherical	*S. aureus*, *E. coli*, *E. faecalis*, *P. aeruginosa* (15–120)	Mechanical destruction of cell wall, cell lysis	Non-cytotoxic	Antibacterial drug	[144]
Dextrose; water solution	Corn	Spherical; 25, 60, 120 nm	*E. coli*	Cell wall structure disruption, cell lysis	-	Antibacterial drug	[132]

**Table 4:** Plant extracts with antibacterial activity.

Plant	Kind of Extract	Extract Composition	Bacterial Strains	Mechanism of Antibacterial Activity	References
*Crotalaria bernieri*	Leaf; hexane extract	Tannins, polyphenols, steroids, triterpenes, unsaturated sterols	*S. aureus*	Unknown	[159]
	Leaf; methanol extract	Alkaloids, flavonoids, tannins, polyphenols	*E. aerogenes*, *P. aeruginosa*, *V. parahaemolyticus*, *P. mirabilis*, *B. cereus*, *C. perfringens*, *S. aureus*, *S. pneumonia*, *S. pyogenes*	Unknown	
	Seed; hexane extract	Tannins, polyphenols, steroids, triterpenes, unsaturated sterols	*V. parahaemolyticus*, *S. aureus*	Unknown	
	Seed; ethyl acetate extract	Flavonoids, tannins, polyphenols, steroids, triterpenes, unsaturated sterols	*E. cloacae*, *S. aureus*, *V. parahaemolyticus*, *P. mirabilis*, *S. pneumoniae*, *S. pyogenes*	Unknown	
	Seed; methanol extract	Flavonoids, tannins, polyphenols, steroids, triterpenes, unsaturated sterols	*Y. enterocolitica*, *S. pneumoniae*, *S. pyogenes*	Unknown	
	Pod; hexane extract	Steroids, triterpenes, unsaturated sterols	*E. aerogenes*, *S. aureus*, *C. perfringens*	Unknown	
	Pod; ethyl acetate extract	Polyphenols, steroids, triterpenes, unsaturated sterols	*V. parahaemolyticus*, *S. aureus*, *B. cereus*, *C. perfringens*, *S. pyogenes*, *S. pneumoniae*	Unknown	
*Crotalaria bernieri*	Pod; methanol extract	Alkaloids, flavonoids	*S. enteridis*, *S. flexneri*, *V. parahaemolyticus*, *P. mirabilit*, *P. mirabilis*, *S. aureus,S. pneumoniae*, *S. pyogenes*	Unknown	[159]
	Root; hexane extract	Tannins, polyphenols, steroids, triterpenes, unsaturated sterols	*P. aeruginosa*, *S. enteridis*, *S. pyogenes*	Unknown	
	Root; ethyl acetate extract	Tannins, polyphenols, steroids, triterpenes, unsaturated sterols	*V. parahaemolyticus*, *B. cereus,C. Perfringens*, *S. aureus*, *S. pneumoniae*, *S. pyogenes*	Unknown	
	Root; methanol extract	Saponins, tannins, polyphenols	*S. enteridis*, *P. mirabilis*, *B. cereus*, *C. perfringens*, *S. aureus*, *S. pneumoniae*, *S. pyogenes*	Unknown	
*Arum maculatum*	Leaf; water extract	Phenols, tannins, tocopherols, flavonoids, beta-carotene	*E. coli*, *S. aureus*, *L. monocytogene*, *S. enteritidis*, *P. aeruginosa*	Unknown	[160]
	Leaf; water: ethanol (50:50) extract				
	Leaf; ethanol extract				
*Luma apiculata*	Leaf; hexane extract	Catechins, flavonoids, glycosyloxyflavone, triterpenoids	*S. aureus*, *S. epidermidis*, *S. saprophyticus*, *E. coli* *A. boumanii*, *P. aeruginosa*, *Enterococcus* sp.	Unknown	[161,162]
	Flower; hexane extract		*S. epidermidis*, *S. aureus*, *S. saprophyticus*, *Enterococcus* sp	Unknown	
Green tea; epigallocatechin 3-gallate	Leaf; water solution	Polyphenol	*S. aureus*, *E. coli*, *E. faecalis*, *P. aeruginosa*	Mechanical destruction of cell wall; cell lysis	[144]
*Areca catechu*	Nuts; water extract	Flavonoids, proteins	*E. cloacae*, *S. haemolyticus*, *S. petrasii*, *B. cereus*, *Enterobacter* sp.	Unknown	[77]
*Salix alba*	Leaf; hydro-alcoholic fraction	Salicin	*S. aureus*, *K. pneumoniae*, *B. sublitis*	Unknown	[106]
*Thymus vulgaris*	Leaf and flower; hydro-alcoholic fraction	Thymol, carvacrol, flavonoids, tannins, triterpenes	*P. aeruginosa*, *Proteus* sp.	Unknown	[158]
*Rosmarinus officinalis*	Leaf; hydro-alcoholic fraction	Flavonoids, phenolic acids (caffeic, chorogenic, rosmarinic), essential oils (camphor, cineole), diterpenes (carnosol)	*B. subtilis*	Unknown	
*Syzygyum joabolanum*	Leaf; hydro-alcoholic fraction	Flavonoids, tannins	*S. aureus*, *K. pneumoniae*	Unknown	
*Punica granatum*	Pericarp; hydro-alcoholic fraction	Ellagitannins, alkaloids	*P. aeruginosa*, *B. subtilis*	Unknown	
*Psidium guajava*	Leaf; hydro-alcoholic fraction	Comarins, essencial oils, flavonoids, triterpenes, ellagitannins	*S. aureus*	Unknown	
*Ocimum* *basilicum*	Leaf; hydro-alcoholic fraction	Essential oils (linalol, estragol, eugenol), tannins, flavonoids	*P. aeruginosa*	Unknown	
*Hibiscus sabdariffa*	Flower; water extract	Phenolic compounds, terpenoids, esters, weak and fatty acids	*E. coli*, *V. parahaemolyticus*, *P. aeruginosa*, *S. enteritidis*, *B. cereus*, *S. aureus*	Membrane potential changes	[72]
	Flower; ethanol extract				
*Syzygium* *aromaticum*	Flower; water extract	Phenolic compounds, terpenoids, esters, weak and fatty acids	*E. coli*, *V. parahaemolyticus*, *P. aeruginosa*, *S. enteritidis*, *B. cereus*, *S. aureus*	Membrane potential changes	[72]
	Flower; ethanol extract				
*Moringa oleifera*	Leaf; ethanol extract	Alkaloids, saponins, tannins, phenolics, flavonoids, triterpenoids, steroids, glycosides	*S. aureus*, *E. coli*	Unknown	[163]
*Magnolia* *acuminata*	Leaf; ethanol extract	Alkaloids, saponins, tannins, phenolics, flavonoids, triterpenoids, steroids, glycosides	*S. aureus*, *E. coli*	Unknown	
*Prunus cerasus*	Leaf; ethanol extract	Alkaloids, saponins, tannins, phenolics, flavonoids, triterpenoids, steroids, glycosides	*S. aureus*, *E. coli*	Unknown	
*Leucaena* *leucocephala*	Leaf; ethanol extract	Alkaloids, saponins, tannins, phenolics, flavonoids, triterpenoids, steroids, glycosides	*S. aureus*, *E. coli*	Unknown	
*Tilia argentea*	Leaf; water extract	Flavonoids, phenolic compounds, esters, terpenes, aliphatic acids, hydrocarbons	*E. coli*, *S. aureus*, *E. pneumoniae*, *B. subtilis*	Unknown	[120,164]
*Anthemis* *pungens*	Leaf; water extract	Phenolic compounds, flavonoids	*E. coli*, *S. aureus*	Unknown	[120]
*Pistacia* sp.	Leaf; water extract	Flavonoids, phenolic compounds, carboxylic acids, aromatic compounds	*E. coli*, *B. subtilis*	Unknown	[120,165]
*Epaltes divaricata*	Whole plant; water extract	Tannins, phenolic compounds, saponins, cardiac glycosides, flavonoids, alkaloids	*S. aureus*	Unknown	[121]
*Asparagus* *falcatus*	Tuber; hexane extract Tuber; ethanol extract	Tannins, phenolic compounds, saponins, cardiac glycosides, flavonoids	*S. aureus*	Unknown	
*Asteracantha longifolia*	Whole plant; hexane extract Whole plant; ethanol extract	Tannins, phenolic compounds, saponins, cardiac glycosides, flavonoids, alkaloids	*S. aureus*	Unknown	
*Vetiveria* *zizanioides*	Root; hexane extract Root; ethanol extract	Tannins, phenolic compounds, saponins, cardiac glycosides, flavonoids, alkaloids	*S. aureus*	High concentration of low-polarity compounds in the extract	
*Coriandrum sativum*	Seed; hexane extract Seed; ethanol extract	Tannins, phenolic compounds, cardiac glycosides, flavonoids, alkaloids	*S. aureus*	Unknown	

## Data Availability

Not applicable.
